# Spinal disinhibition: evidence for a hyperpathia phenotype in painful diabetic neuropathy

**DOI:** 10.1093/braincomms/fcad051

**Published:** 2023-02-28

**Authors:** Anne Marshall, Alise Kalteniece, Maryam Ferdousi, Shazli Azmi, Edward B Jude, Clare Adamson, Luca D’Onofrio, Shaishav Dhage, Handrean Soran, Jackie Campbell, Corinne A Lee-Kubli, Shaheen Hamdy, Rayaz A Malik, Nigel A Calcutt, Andrew G Marshall

**Affiliations:** Division of Diabetes, Endocrinology and Gastroenterology, Faculty of Biology, Medicine and Health, University of Manchester, Manchester M13 9PL, UK; Institute of Life course and Medical Sciences, University of Liverpool, Liverpool L69 3BX, UK; Division of Cardiovascular Sciences, Faculty of Biology, Medicine and Health, University of Manchester, Manchester M13 9PL, UK; Division of Cardiovascular Sciences, Faculty of Biology, Medicine and Health, University of Manchester, Manchester M13 9PL, UK; Division of Cardiovascular Sciences, Faculty of Biology, Medicine and Health, University of Manchester, Manchester M13 9PL, UK; Diabetes Centre, Manchester University NHS Foundation Trust, Manchester M13 9WL, UK; Division of Diabetes, Endocrinology and Gastroenterology, Faculty of Biology, Medicine and Health, University of Manchester, Manchester M13 9PL, UK; Department of Diabetes and Endocrinology, Tameside and Glossop Integrated Care NHS Foundation Trust, Manchester OL6 9RW, UK; Diabetes Centre, Manchester University NHS Foundation Trust, Manchester M13 9WL, UK; Department of Experimental Medicine, Sapienza University, Rome 00185, Italy; Division of Cardiovascular Sciences, Faculty of Biology, Medicine and Health, University of Manchester, Manchester M13 9PL, UK; Division of Cardiovascular Sciences, Faculty of Biology, Medicine and Health, University of Manchester, Manchester M13 9PL, UK; Faculty of Health, Education and Society, University of Northampton, Northampton NN1 5PH, UK; Molecular Neurobiology Laboratory, The Salk Institute for Biological Studies, La Jolla, CA 92037, USA; Division of Diabetes, Endocrinology and Gastroenterology, Faculty of Biology, Medicine and Health, University of Manchester, Manchester M13 9PL, UK; Division of Cardiovascular Sciences, Faculty of Biology, Medicine and Health, University of Manchester, Manchester M13 9PL, UK; Weill Cornell Medicine-Qatar, Research Division, Qatar Foundation, Doha 24144, Qatar; Department of Pathology, University of California, La Jolla, CA 92093, USA; Institute of Life course and Medical Sciences, University of Liverpool, Liverpool L69 3BX, UK; Division of Neuroscience and Experimental Psychology, Faculty of Medical and Human Sciences, University of Manchester, Manchester M13 9PL, UK

**Keywords:** diabetes, neuropathy, pain, spinal disinhibition, phenotype

## Abstract

The dominant sensory phenotype in patients with diabetic polyneuropathy and neuropathic pain is a loss of function. This raises questions as to which mechanisms underlie pain generation in the face of potentially reduced afferent input. One potential mechanism is spinal disinhibition, whereby a loss of spinal inhibition leads to increased ascending nociceptive drive due to amplification of, or a failure to suppress, incoming signals from the periphery. We aimed to explore whether a putative biomarker of spinal disinhibition, impaired rate-dependent depression of the Hoffmann reflex, is associated with a mechanistically appropriate and distinct pain phenotype in patients with painful diabetic neuropathy. In this cross-sectional study, 93 patients with diabetic neuropathy underwent testing of Hoffmann reflex rate-dependent depression and detailed clinical and sensory phenotyping, including quantitative sensory testing. Compared to neuropathic patients without pain, patients with painful diabetic neuropathy had impaired Hoffmann reflex rate-dependent depression at 1, 2 and 3 Hz (*P* ≤ 0.001). Patients with painful diabetic neuropathy exhibited an overall loss of function profile on quantitative sensory testing. However, within the painful diabetic neuropathy group, cluster analysis showed evidence of greater spinal disinhibition associated with greater mechanical pain sensitivity, relative heat hyperalgesia and higher ratings of spontaneous burning pain. These findings support spinal disinhibition as an important centrally mediated pain amplification mechanism in painful diabetic neuropathy. Furthermore, our analysis indicates an association between spinal disinhibition and a distinct phenotype, arguably akin to hyperpathia, with combined loss and relative gain of function leading to increasing nociceptive drive.

## Teaser

Marshall *et al.* report that patients with painful diabetic neuropathy and evidence of spinal disinhibition, demonstrated by impairment of Hoffman reflex rate-dependent depression, display a mechanistically relevant pain phenotype. This phenotype, akin to hyperpathia, exhibits a combined loss and relative gain of function leading to increased nociceptive drive.

## Graphical Abstract

**Graphical Abstract:**
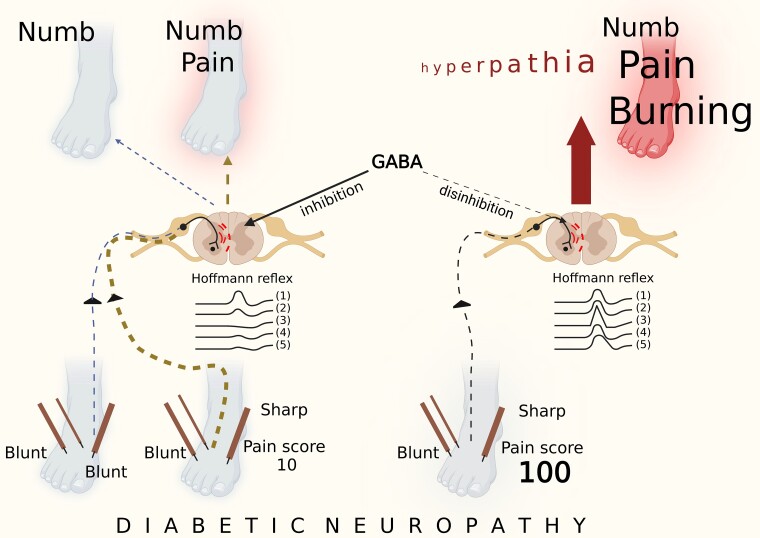

## Introduction

Diabetic peripheral neuropathy (DPN) is characterized by an array of ‘negative’ and ‘positive’ sensory symptoms in which numbness may paradoxically coexist with prickling, stabbing, burning or aching pain.^1^ These painful sensations may occur in response to normally innocuous stimuli (allodynia), because of increased sensitivity to painful stimuli (hyperalgesia) or arise spontaneously. The symptoms that dominate can vary dramatically between patients, and it is not yet established whether signs and symptoms change with progression of neuropathy.^2^ The recent systematic application of sensory phenotyping using quantitative sensory testing (QST) has enabled stratification of patients with DPN into clusters of characteristics and has been proposed to potentially segregate patients based on underlying physiological mechanisms.^3-6^

It has been widely argued that ‘dying back’ nerve fibre degeneration and/or nerve fibre regeneration may contribute to the development of pain in diabetic and other forms of neuropathic pain by causing nociceptors to become abnormally spontaneously active or to become sensitized (i.e. respond more vigorously to a given stimulus or to a lower strength of stimulus).^7-10^ These features could contribute to spontaneous pain, such as burning pain, as well as hyperalgesia and allodynia. However, preclinical studies have reported diminished release of excitatory neurotransmitters in the spinal cord of diabetic rats during periods of stimulus-evoked behavioral hyperalgesia,^11,12^ and QST studies have reported that only a small proportion of patients with painful DPN fit into the ‘irritable nociceptor’ sensory phenotype.^3,4,13^ Indeed, loss of function consistent with a ‘deafferentation’ phenotype is the most commonly demonstrated QST profile in patients with DPN.^3,4,13^ Whilst variability exists within and across these broad sensory phenotypes, suggesting diversity of pain generation or modulation mechanisms, it remains unclear as to how a deafferentation phenotype, with apparent loss of function of nociceptive pathways, leads to pain.

It is increasingly accepted that pro-nociceptive pathophysiological changes occur within the spinal cord in diabetes that could generate or maintain pain. Temporal summation of pain (wind-up)^14^ or alterations in descending pain modulation^15-17^ have been investigated. Whilst imaging studies suggest enhanced descending facilitation,^17^ evidence of these mechanisms in pain generation in patients with DPN has been inconsistent.^4,18,19^ A further potential mechanism of interest is spinal disinhibition, whereby inappropriate amplification of, or failure to suppress, incoming signals from the periphery leads to facilitation of ascending nociceptive drive—a process that could generate pain despite peripheral loss of function. In diabetic rodents, spinal disinhibition results from a brain-derived neurotrophic factor-dependent reduction in the expression of potassium chloride co-transporter 2 in the dorsal horn of the spinal cord, leading to a shift in the function of ionotropic GABA-A receptors from inhibitory towards excitatory.^20^ A biomarker for spinal disinhibition in diabetic rodents is impaired Hoffmann reflex rate-dependent depression (HRDD).^21^ Importantly, interventions targeting the underlying mechanisms of spinal disinhibition in rats both normalize impairments in HRDD and ameliorate behavioral manifestations of pain.^22^ We have recently translated these experimental findings to the clinical setting by demonstrating impairment of HRDD in subjects with painful DPN,^22,23^ indicating that spinal disinhibition may be a dominant pain mechanism in a proportion of these patients.

It is currently unknown whether patients with painful DPN and impaired HRDD have a distinct pain phenotype reflecting spinal disinhibition or whether impairment of HRDD is associated with other mechanisms facilitating ascending spinal nociceptive information such as wind-up or impaired descending pain modulation. To further investigate the relationship between spinal disinhibition and pain phenotype, we have explored QST somatosensory profiles and conditioned pain modulation in conjunction with HRDD in a cohort of patients with diabetes, with and without neuropathic pain.

## Materials and methods

This was an observational cross-sectional study. Research Ethics Committee approval was granted (East Midlands—Leicester South Research Ethics Committee reference 17/EM/0076), and written informed consent was obtained from each participant. Study conduct adhered to the tenets of the Declaration of Helsinki. Consecutive patients attending secondary care diabetes clinics at Manchester University NHS Foundation Trust and Tameside and Glossop Integrated Care NHS Foundation Trust between November 2017 and February 2020 were invited to take part in the study. Participants underwent assessment during a single research visit.

### Study participants

Ninety-three patients with type 1 or type 2 diabetes were recruited into the study. The majority (*n* = 90/93) were recruited from a previously reported cohort.^23,24^ Detailed demographic data including age, gender and ethnicity along with type and duration of diabetes, co-morbidities, medication, height, weight, blood pressure, HbA1c, lipids and renal function were documented. Participants found to have other common causes of neuropathy based on a family history as well as testing for serum B_12_, folate, immunoglobulins, electrophoresis and anti-nuclear antibody were excluded from the study.

### Neuropathy and pain questionnaires

Participants completed five questionnaires. The Neuropathy Symptom Profile (NSP), a yes or no questionnaire that documents sensory, autonomic and motor symptoms, including weakness, has been validated in patients with DPN^25^ and found to be particularly useful in recognizing patterns of symptoms. The Small Fibre Neuropathy and Symptom Inventory Questionnaire (SFN-SIQ) was used to assess the presence of sensory and autonomic symptoms including changes in sweating patterns, diarrhoea, constipation, urinary tract problems, dry eyes, dry mouth, dizziness, hot flushes, palpitations, sensitive leg skin, restless legs, burning feet and sheet intolerance. The Diabetic Neuropathy Symptom Score (DNS) was used as a diabetes-specific simplified scoring system assessing pain, numbness, tingling and ataxia^26^ with any score above 0 representing an abnormality. The Neuropathy Pain Scale (NPS), a 0-10 pain rating scale, was used to define the severity of symptoms based on patient responses to questions about pain intensity and pain descriptors, for example, sharp, dull, hot, cold, skin sensitivity and itch. Participants were asked to mark on three visual analogue scales (VAS) current pain, average pain over the past 24 h and worst pain over the past 24 h.

### Nerve conduction and H-reflex studies

**Figure 1:**
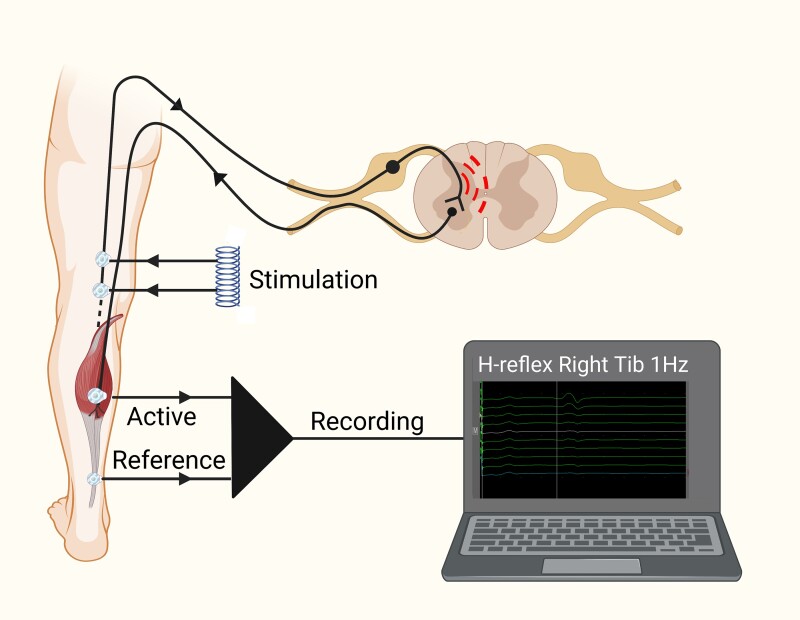
**A schematic representation for eliciting and recording the H-reflex** (Created with BioRender.com).

Nerve conduction and H-reflex studies were performed using a DANTEC Keypoint system (Dantec Dynamics Ltd., Bristol, UK). Participants were semi-recumbent at 45° with limb temperature maintained between 32° and 35°. Sural sensory amplitude and conduction velocity along with peroneal motor nerve amplitude and conduction velocity were recorded. For H-reflex studies, tibial nerve stimulation was performed using 1-ms square wave monophasic pulses delivered using surface silver–silver chloride electrodes, to the popliteal fossa. Surface silver–silver chloride recording electrodes with a diameter of 9 mm were placed on the long axis of soleus (Fig. 1). H-reflex recruitment curves were obtained to determine peak–peak H-reflex maximal amplitude by incrementing stimulation current by 1 mA (1-ms duration). A random inter-stimulation interval with a minimum of 10 s was observed. For HRDD, a submaximal stimulus strength (to achieve a response of 75% of maximum H-reflex on the rising phase of the recruitment curve) was used. H-wave responses were recorded in trains of ten stimuli delivered at 1–3 Hz. HRDD was calculated as the mean H-reflex amplitude of responses 2–5 of a stimulus train, expressed as a percentage of the amplitude of the first recorded H-reflex in the train. Therefore, a higher value of HRDD indicates a smaller degree of depression than a lower value and vice versa. The average of stimulus responses 2–5 was used as this has been shown to be the optimal value to discriminate between patients with painful and painless DPN.^24^

### Corneal confocal microscopy

Corneal confocal microscopy (CCM) was used to quantify corneal small nerve fibre pathology and has been validated against the current gold standard of intraepidermal nerve fibre density.^27^ Images of the corneal sub-basal nerve plexus were captured using the Heidelberg Retina Tomograph 3 with Rostock Cornea Module (Heidelberg Eye Explorer, Heidelberg Engineering GmBH, Heidelberg, Germany) following an established protocol.^28^ For image analysis, six representative images (three per eye) were selected by M.F. and A.K., who were blinded to participant status. Corneal nerve fibre density [total number of main nerves per square millimetre (no./mm^2^)], corneal nerve fibre length [total length of main nerves and nerve branches per square millimetre (mm/mm^2^)] and corneal nerve branch density [total number of branches per square millimetre (no./mm^2^)] were quantified.

### Quantitative sensory testing

A full QST battery, representing seven tests assessing 13 parameters, was performed on all patients with diabetes using the standardized DFNS testing protocol.^29^ The investigators (A.M. and A.G.M.) underwent formal training at the University of Mannheim prior to commencing this study. Tests for thermal sensation were performed at the beginning of the testing paradigm, prior to mechanical assessments. The thermal sensory testing device (TSA-II NeuroSensory Analyser Medoc, Ltd., Ramat-Yishai, Israel) was positioned on the skin on the dorsum of the right foot. Cold detection threshold (CDT) and warm detection threshold (WDT) along with cold pain threshold (CPT) and heat pain threshold (HPT) were recorded. The threshold was determined as the arithmetic mean of three results using the difference between measured threshold and baseline temperature (32°C) for CDT and WDT and absolute temperature for CPT and HPT. Testing of the thermal sensory limen was also performed and calculated subtracting the arithmetic mean of the CDT from the arithmetic mean of the WDT. Paradoxical heat sensations were recorded. Mechanical detection threshold was assessed using standardized Von Frey hairs (0.25, 0.5, 1, 2, 4, 8, 16, 32, 64, 128, 256 and 512 mN Opti-hair2-Set, Marstock Nervtest, Germany) and calculated using a modified method of limits (geometric mean of five supra and subthreshold stimulus responses). Mechanical pain threshold (MPT), mechanical pain sensitivity (MPS) and wind-up ratio were all assessed using a set of seven pinprick stimulators with standardized intensities (8, 16, 32, 64, 128, 256 and 512 mN). MPT was calculated using a modified method of limits (geometric mean of five supra and subthreshold stimulus responses). The degree of MPS was calculated using the geometric mean of pain ratings for pinprick stimuli, and wind-up ratio was calculated as the arithmetic mean of the pain intensity rating for the series of stimuli divided by the arithmetic mean of the pain intensity rating for the single stimulus. Dynamic mechanical allodynia, the degree of pain sensitivity to innocuous stimuli, was assessed on the dorsum of the right foot using a cotton wisp (exerting a force of 3 mN), a Q-tip (exerting a force of 100 mN) and a soft brush (exerting a force of between 200 and 400 nM), applied in a balanced order and pain ratings recorded. Dynamic mechanical allodynia was calculated as geometric mean of pain ratings. Vibration detection thresholds were recorded using a tuning fork (Rydel Seiffer 64 Hz with fixed weights) over the medial malleolus with the threshold determined by the arithmetic mean of the three values. Pressure pain thresholds were recorded using a pressure algometer (FDN200, Wagner Instruments, USA) with a blunt contact area of 1 cm² placed on the skin above the abductor hallucis muscle. The threshold was determined as the arithmetic mean of the three recordings. The raw QST data from each test were log transformed and converted into *z*-scores (with exception of paradoxical heat sensations and dynamic mechanical allodynia) to normalize the data for age, sex and body site tested. This transformation enables comparison between cohorts and DFNS reference data^29^ and allows for the identification of specific QST profiles. Positive *z*-score values denote a gain in function, and negative *z*-scores denote a loss of function in each of the parameters.

We calculated a value for mechanical pain differential as (*z*-score for MPS) **−** (*z*-score for MPT). This pain differential (MPS-MPT) gave us a value that represents the ‘relative’ gain and loss of function for mechanical pinprick. A high pain differential score represents patients who have a high MPS ‘relative’ to MPT. We also calculated a value for thermal pain differentials: (*z*-score for CPT) − (*z*-score for CDT) termed CPT-CDT and (*z*-score for HPT) − (*z*-score for WDT) termed HPT-WDT.

### Conditioned pain modulation

Conditioned pain modulation requires intact descending pathways and has been shown to be attenuated in patients with chronic pain.^15,30^ Pressure pain threshold on the right abductor pollicis brevis was used as the test stimulus. A pressure algometer (FDN200, Wagner Instruments, USA) with a blunt contact area of 1 cm² was placed on the skin above the abductor hallucis muscle on the right hand. Pressure was applied with increasing intensity at a rate of 0.5 kg (50 kPa)/s. The patient was asked to indicate as soon as the sensation of pressure changed to an additional painful ‘burning’, ‘stinging’ or ‘aching’ sensation and the value on the algometer recorded. The test was repeated three times with a break of 10 s in between and mean value recorded. A conditioning stimulus using noxious cold was then administered. The left hand of the patient was immersed up to the wrist in a water bath of melting ice water for up to 180 s. The patient was asked to rate how painful this was (0–100) every 15 s. When the patient could no longer tolerate it, their hand was removed from the water bath and the time noted. The test stimuli were then repeated on the right hand (non-submerged) as detailed above and the level of pain intensity rated again. The conditioned pain modulation effect was calculated as the difference (post conditioning stimulus minus pre) in pressure pain thresholds. A positive value indicates efficient conditioned pain modulation.

### Statistical methods

Statistical analyses were performed using Prism 9 statistical software (GraphPad Software Inc, La Jolla, CA, USA) and IBM SPSS 29 (cluster analysis). Data were tested for normality with the Shapiro–Wilk test of normality. Categorical data were analysed using chi-square Fisher’s exact test of association. Parametric data were analysed using unpaired *t*-test to compare means between two groups. Results were reported as mean ± standard deviation. Non-parametric data were analysed using Mann–Whitney test between two groups. Results were reported as median with interquartile range. A *P*-value of <0.05 considered significant. Correlations were performed using Spearman’s rank test and expressed as a coefficient (*r*) with *P*-values. A Bonferroni correction was applied to account for multiple comparisons resulting in a significant *P*-value of 0.0016. A *k*-means clustering algorithm (SPSS) was used to further investigate associations within the pain cohort, grouping the data set based on HRDD, QST parameters and pain descriptors from the NPS questionnaire.

## Results

A total of 93 patients with DPN, 37 with neuropathic pain and (VAS > 0) and 56 without neuropathic pain (VAS = 0) were recruited. Within the painful DPN group, seven patients were taking medication to treat neuropathic pain (2 × duloxetine, 3 × gapapentinoids and 2 × tricyclics). An additional five patients were taking selective serotonin reuptake inhibitors. Coincidentally, within the painless DPN group, two patients were taking tricyclics. Current, average and maximum pain scores did not differ significantly between patients with type 1 or type 2 diabetes. There was no significant difference for age, gender, ethnicity, body mass index (BMI) and type or duration of diabetes between the pain and no pain cohorts (Table 1). The HbA1c was significantly (*P* = 0.018) higher in patients with painless DPN compared to patients with painful DPN.

**Table 1:** Demographic and neuropathy parameters for patients with DPN, with and without neuropathic pain

	DPN with pain (*n* = 37)	DPN without pain (*n* = 56)
Type of diabetes (1/2)	11/26	21/35
Gender (female/male)	17/20	19/37
Ethnicity (White/Asian/Black)	28/7/2	41/12/3
	Median (interquartile range)	
Age (years)	62 (53–71.5)	65 (52.5–71)
Duration (years)	15 (9–22)	16 (10–23)
HbA1c (mmol/mol)	53.5 (46.6–57.3)*	58.0 (34.0–69.0)
BMI (kg/m²)	29.2 (25.5–31.8)	27.7 (24.6–32)
NSP	5.0 (3.25–10)***	2.0 (0.5–3.5)
SNAP (µV)	7.5 (3–15.5)	6.8 (4–11.8)
SNCV (m/s)	41.2 (38.9–47.9)	43.1 (40–46.7)
PMNAP (mV)	3.6 (2.3–5.6)	3.5 (2.5–5.4)
PMNCV (m/s)	41.2 (37.4–43.5)	40.9 (38.6–44.1)
CNFD (no./mm²)	24.48 (18.49–28.39)	26.04 (18.75–30.21)
CNFL (mm/mm²)	17.75 (13.58–21.25)	16.93 (13.33–21.25)
CNBD (no./mm²)	49.48 (31.51–87.5)	40.62 (23.96–58.85)
VAS pain current (0–100)	14.0 (4.25–30.75)	0
VAS pain av past 24 h	35.5 (15.5–65.5)	0
VAS pain max past 24 h	51.0 (26.5–76.5)	0
	Mean ± SD	
HRDD mean H2–5 @ 1 Hz	64.83 ± 22.60***	36.42 ± 16.69
HRDD mean H2-5 @ 2 Hz	52.51 ± 28.28***	30.41 ± 15.31
HRDD mean H2-5 @ 3 Hz	53.00 ± 26.19***	28.22 ± 14.32

Categorical data were analysed by chi-square (Fisher’s exact) test. Non-parametric data were shown as median (interquartile range) and analysed by the Mann–Whitney test. Parametric data were shown as mean ± SD and analysed using unpaired *t*-test. BMI, body mass index; CNBD, corneal nerve branch density; CNFD, corneal nerve fibre density; CNFL, corneal nerve fibre length; HRDD, Hoffmann reflex rate-dependent depression; NSP, Neuropathy Symptom Profile; PMNAP, peroneal motor nerve amplitude; PMNCV, peroneal motor nerve conduction velocity; SNAP, sural nerve amplitude; SNCV, sural nerve conduction velocity; VAS, Visual Analogue Scale. *P≤0.05, ***P≤0.001.

### Neuropathy assessments and questionnaires

**Figure 2:**
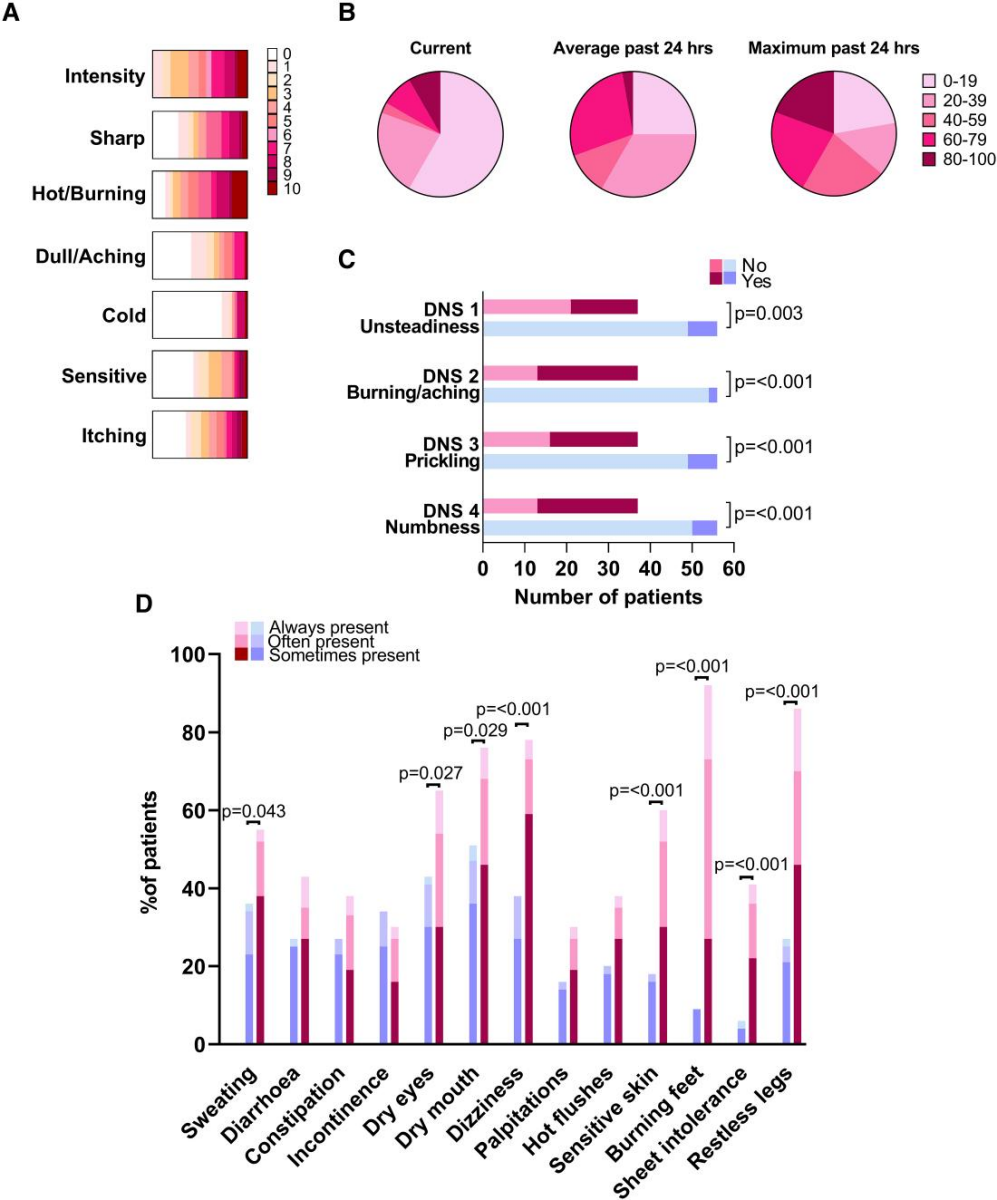
**Pain scales and descriptors.** (**A**) NPS in patients with painful DPN. The coloured bars represent the distribution of patient responses for each score (0–10). (**B**) VAS pain score in patients with painful DPN for current, average and maximum pain during the past 24 h. The coloured segments represent the proportion of patient responses within each category. (**C**) Diabetic Neuropathy Symptom Score in patients with DPN with (magenta bars) and without (blue bars) neuropathic pain. (**D**) SFN-SIQ in patients with DPN with (magenta bars) and without (blue bars) neuropathic pain. Statistically significant *P*-values shown (Mann–Whitney U test).

There were no significant differences in data obtained from nerve conduction studies and corneal confocal microscopy between the cohorts of patients with and without neuropathic pain. Of all subjects studied, 22 patients (10 with neuropathic pain and 12 without neuropathic pain) had both nerve conduction parameters within local normative values and CCM parameters within the previously published normative range.^31^Figure 2A and B show the distribution of descriptor ratings on the NPS and VAS pain scores reported by patients with painful DPN. Figure 2C shows the number of patients, with DPN with and without pain, reporting neuropathy symptoms on the DNS. The NSP, DNS and SFN-SIQ were significantly (all *P* < 0.001) higher in patients with neuropathic pain compared to those without pain. Symptoms related to pain and hypersensitivity represented the highest proportion of divergent scores between the two groups, with dry eyes, dry mouth, changes in sweating and dizziness on standing increased in the pain cohort (Fig. 2D). Of note, a small number of patients in the group without neuropathic pain reported burning sensation, sensitive skin, sheet intolerance and restless legs (Fig. 2D) that were not described as painful by these patients.

### Painful and painless DPN is associated with a loss of function sensory profile

**Figure 3:**
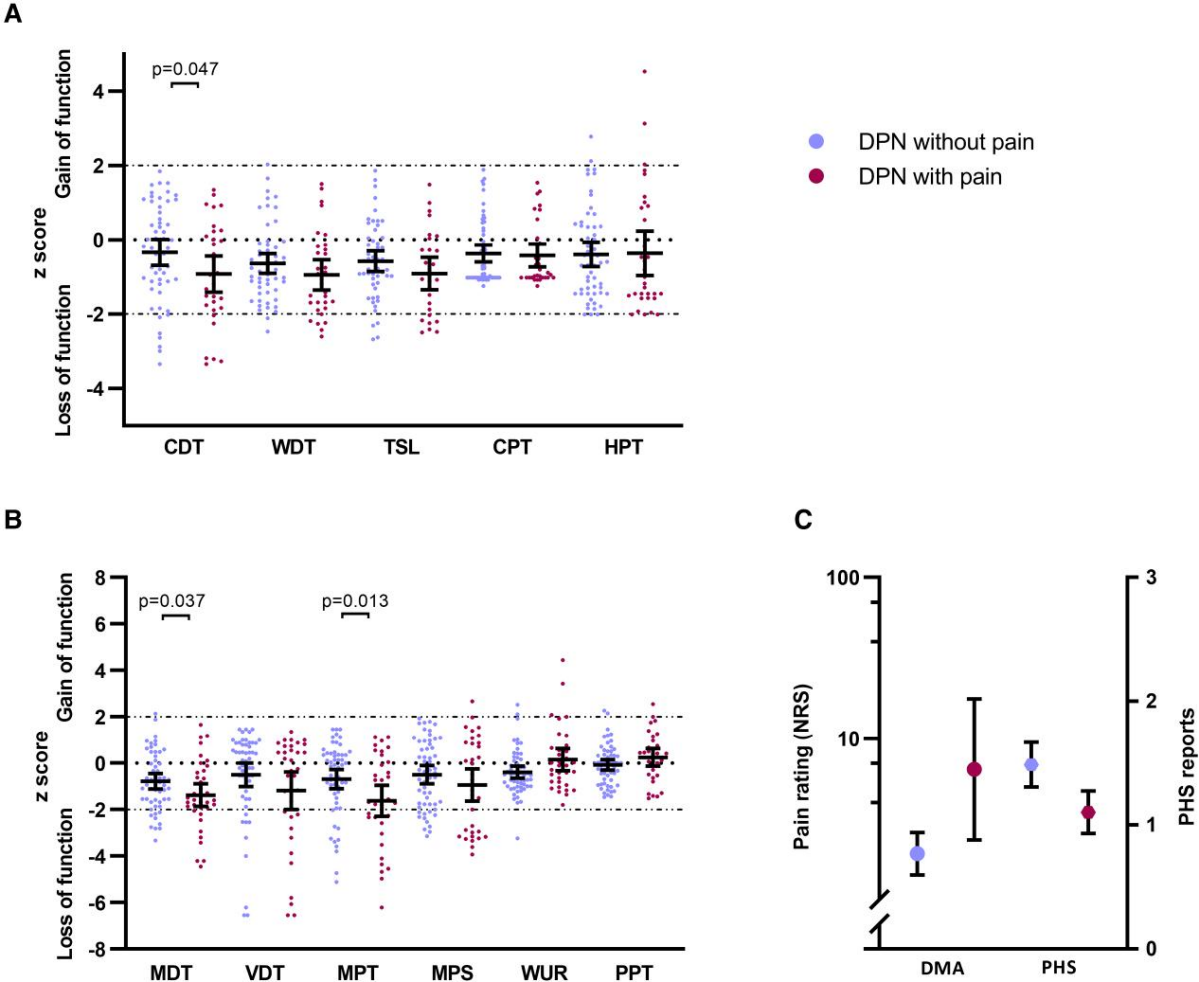
**Sensory profile.** (**A**) Scatter plot and mean ± 95% confidence interval (CI) of *z*-scores for thermal quantitative sensory testing parameters in patients with DPN with (magenta dots) and without (blue dots) neuropathic pain. (**B**) Scatter plot and mean ± 95% CI of *z*-scores for mechanical quantitative sensory testing parameters in patients with DPN with (magenta dots) and without (blue dots) neuropathic pain. (**C**) Dynamic mechanical allodynia and paradoxical heat sensations in patients with DPN with (magenta dot) and without (blue dot) neuropathic pain. Statistically significant *P*-values shown (Mann–Whitney U test). CDT, cold detection threshold; CPT, cold pain threshold; DMA, dynamic mechanical allodynia; HPT, heat pain threshold; MDT, mechanical detection threshold; MPS, mechanical pain sensitivity; MPT, mechanical pain threshold; PHS, paradoxical heat sensation; PPT, pressure pain threshold; TSL, thermal sensory limen; VDT, vibration detection threshold; WDT, warm detection threshold; WUR, wind-up ratio.

**Figure 4:**
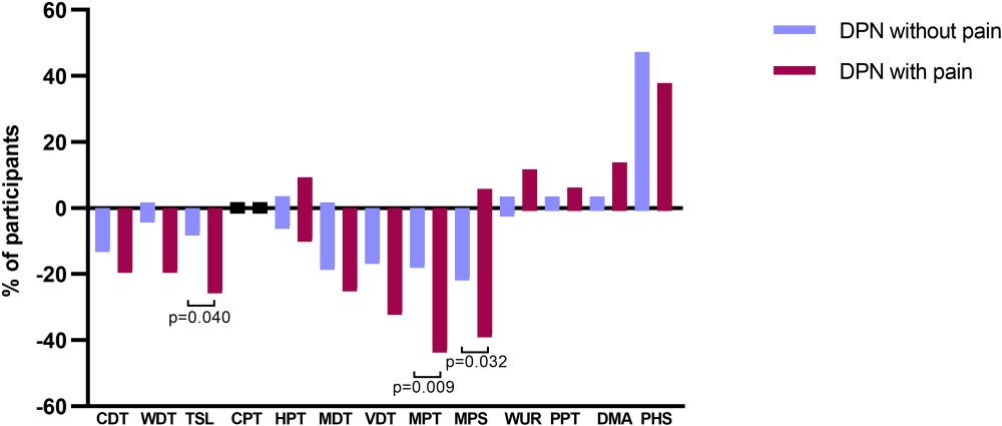
**Loss and gain of sensory function.** Comparison of patients with DPN with (magenta) and without (blue) neuropathic pain who have QST values outside the 95% confidence interval of the German research network of neuropathic pain reference database. Statistically significant *P*-values shown (Fisher’s exact test). CDT, cold detection threshold; CPT, cold pain threshold; DMA, dynamic mechanical allodynia; HPT, heat pain threshold; MDT, mechanical detection threshold; MPS, mechanical pain sensitivity; MPT, mechanical pain threshold; PHS, paradoxical heat sensation; PPT, pressure pain threshold; TSL, thermal sensory limen; VDT, vibration detection threshold; WDT, warm detection threshold; WUR, wind-up ratio.

Individual *z*-scores for QST parameters are summarized in Fig. 3A and B. Whilst the mean *z*-score for all parameters fell within the normative range of DFNS control data,^29,32^ there was evidence of a loss of function for innocuous and noxious thermal and mechanical detection thresholds as well as for MPS. The *z*-scores for wind-up ratio and pressure pain threshold were within the normative range. Figure 4 shows the proportion of patients with *z*-scores outside the DFNS normative range. Approximately 20% of patients in both the painful and painless DPN cohorts exhibited abnormal loss of function (*z*-score > −1.96) in mechanical detection and MPT. A smaller number (∼10%) from both cohorts showed abnormal loss of function in thermal detection thresholds. A small minority of patients from both cohorts exhibited a gain in function (*z*-score > +1.96) in CPT, HPT, MPS, wind-up ratio and pressure pain threshold. The painful DPN cohort showed a significantly greater loss of function of CDT (*P* = 0.047), mechanical detection threshold (*P* = 0.037) and MPT (*P* = 0.013) compared to patients with painless DPN indicating greater cold and mechanical hypoesthesia. Dynamic mechanical allodynia was present to a greater extent in patients with neuropathic pain but did not reach a level of significance (Fig. 3C). There was no significant difference in the presence of paradoxical heat sensations between the two groups (Fig. 3C). The level of conditioned pain modulation did not differ significantly between the two groups.

Across the whole cohort, age was negatively correlated with thermal, mechanical and vibration detection *z*-scores, even after adjustment for the duration of diabetes, indicating a loss of function with increasing age in patients with diabetes that was over and above the *z*-score transformation to account for age. A greater loss of function of thermal and mechanical detection parameters was associated with increasing large and small fibre neuropathy.

### Patients with painful DPN show impaired HRDD

**Figure 5:**
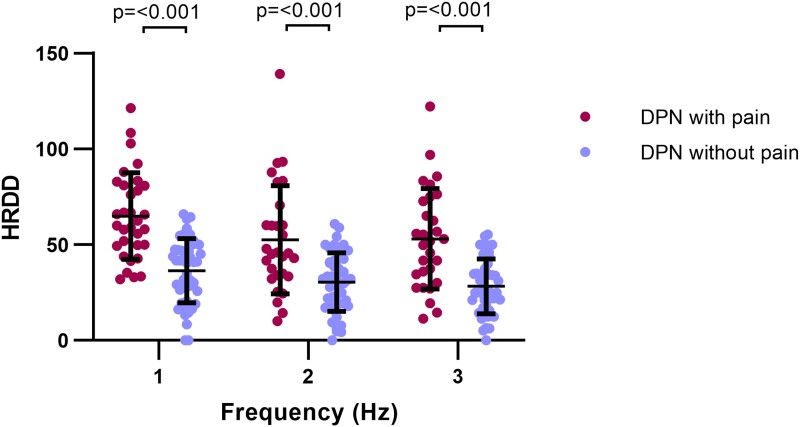
**H-reflex rate-dependent depression.** HRDD at 1, 2 and 3 Hz in patients with DPN with (magenta) and without (blue) pain. Statistically significant *P*-values shown (unpaired *t*-test).

HRDD recordings were available from 82 patients (Supplementary Fig. 1) as 11 patient recordings were incomplete or had technically compromised stimulus response trains. Between group analysis of HRDD (Fig. 5) was consistent with our previously reported findings.^23^ Thus, HRDD was significantly impaired in patients with painful DPN when compared to patients with painless DPN at 1, 2 and 3 Hz (all *P* ≤ 0.001) (Fig. 5). Amongst all patients with DPN (*n* = 82), there were no significant correlations between HRDD and individual QST *z*-scores or with conditioned pain modulation (Supplementary Table 1).

### Increasing impairment of HRDD is associated with relative thermal and mechanical hyperalgesia

Patients with painful DPN demonstrated significantly (*P* = 0.013) greater loss of function in MPT compared to patients with painless DPN, indicating that patients with painful DPN required a stronger stimulus to feel pinprick as painful compared to patients with painless DPN. MPS did not differ significantly between the painful and painless DPN groups. However, patients with the most impaired HRDD amongst those with painful DPN also showed the most gain, or least loss, of function in mechanical pain reporting (Supplementary Table 2).

**Figure 6:**
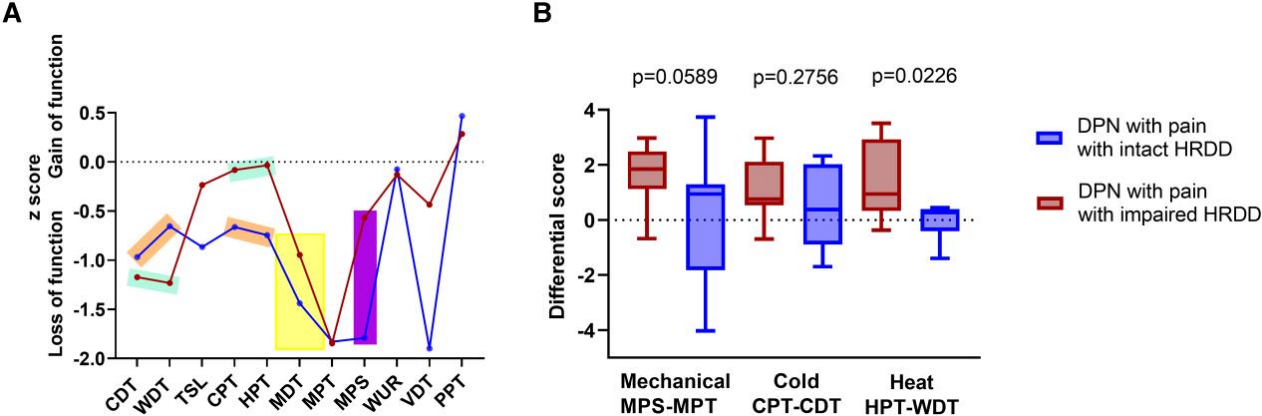
**Hyperpathia profile in patients with painful DPN and impaired HRDD.** (**A**) QST profile for patients with painful DPN and impaired HRDD (red circles/line) and patients with painful DPN and intact HRDD (blue circles/line). Orange boxes highlight *z*-scores for thermal detection and pain thresholds in patients with painful DPN and intact HRDD. Aqua boxes highlight *z*-scores for thermal detection and pain thresholds in patients with painful DPN and impaired HRDD. Yellow box highlights *z*-scores for mechanical pain threshold and sensitivity in both patients with painful DPN with intact and impaired HRDD. Purple box highlights *z*-scores for vibration detection thresholds in both patients with painful DPN with intact and impaired HRDD. (**B**) Mechanical and thermal pain differentials in patients with painful DPN and impaired HRDD (red) and intact HRDD (blue). *P*-values are shown (unpaired *t*-test). CDT, cold detection threshold; CPT, cold pain threshold; HPT, heat pain threshold; MDT, mechanical detection threshold; MPS, mechanical pain sensitivity; MPT, mechanical pain threshold; PPT, pressure pain threshold; TSL, thermal sensory limen; VDT, vibration detection threshold; WDT, warm detection threshold; WUR, wind-up ratio.

To further investigate differences in sensory phenotypes within the pain cohort that could reflect spinal disinhibition as a dominant pain mechanism, we divided patients according to their HRDD status: those with HRDD above 2 SD of the mean of patients with DPN and no pain (*n* = 11) and an equivalent number of patients with painful DPN and the most efficient HRDD. This approach was used as no patients with painful DPN demonstrated efficient HRDD outside 2 SD of patients with painless DPN. Figure 6A shows the QST profiles of these two cohorts of patients (see also Supplementary Table 3). Thermal detection and thermal pain thresholds for patients with the most efficient HRDD showed a similar degree of loss of function (Fig. 6A orange boxes). In contrast, patients with impaired HRDD demonstrated relatively less loss of function in thermal pain thresholds (Fig. 6A, aqua boxes). Therefore, patients with impaired HRDD require a greater temperature change to initially detect heat/cold, but once perceived, it rapidly becomes painful. The mean *z*-score for MPT was comparable in both groups; most patients exhibited loss of function with reduced ability in detecting a sharp sensation. Patients in the pain group with relatively unimpaired HRDD also demonstrated a large loss of function in MPS. However, in patients in the pain cohort with the most impaired HRDD, MPS was relatively preserved with a less pronounced loss of function, but without a gain of function (Fig. 6A, yellow box). In a separate analysis, patients amongst all groups were also divided into two cohorts: HRDD above mean +2 SD of the control group (*n* = 14) and HRDD below mean −2 SD of the control group (*n* = 11), using our previously published normative data from healthy control participants.^24^ This resulted in similar findings (Supplementary Fig. 2A).

Although not statistically significant, these findings led us to explore the difference between the first sensation of sharpness/pain and, once felt, the pain scores attributed to this sharpness as well as the difference in thermal pain and detection thresholds for cold and heat.

### Mechanical pain differential = mechanical pain sensitivity − mechanical pain threshold (MPS-MPT)

This pain differential gives a value that represents the ‘relative’ gain and loss of function for mechanical pinprick. A high pain differential score represents patients who have a high MPS ‘relative’ to their MPT.

There was no significant difference in MPS-MPT between the pain and no-pain groups. However, MPS-MPT was significantly (*P* = 0.023) higher in patients displaying the most impaired HRDD compared to those with the most efficient HRDD (Supplementary Fig. 2B).

**Figure 7:**
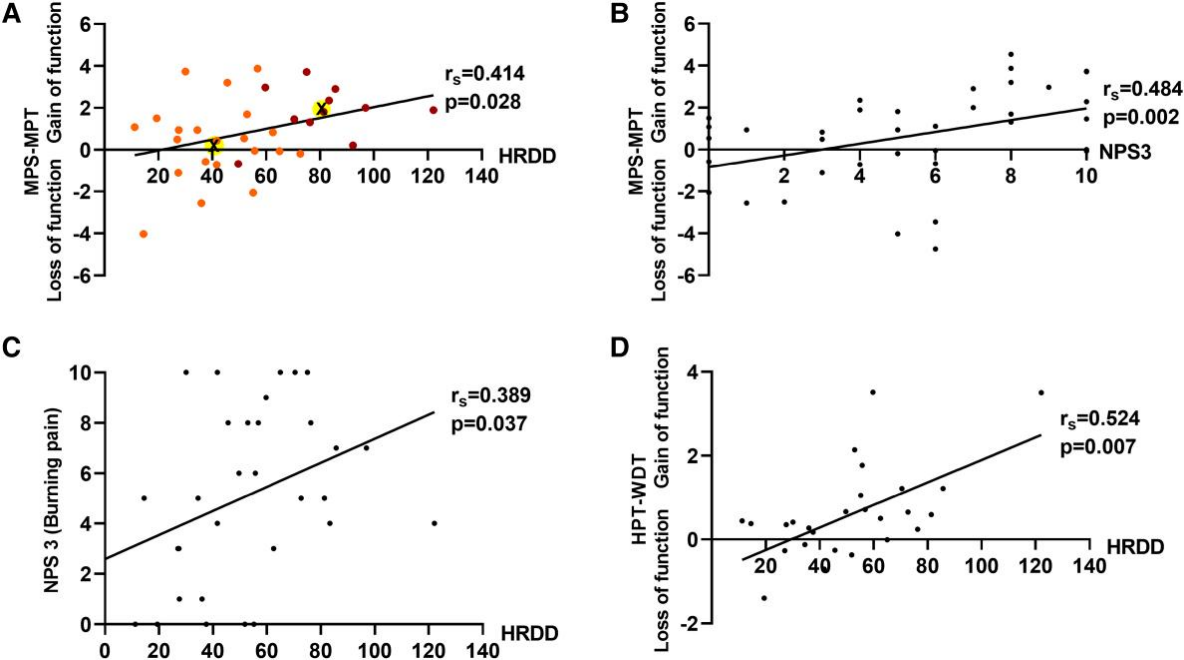
**Correlation graphs.** Scatterplots demonstrating hyperpathia (**A–D**) phenotype in patients with DPN. (**A**) Mechanical pain differential (MPS-MPT) and H-reflex rate-dependent depression [HRDD (H2–5)] at 3 Hz. Red dots: Cluster 1. Orange dots: Cluster 2. X in highlighted circles indicates centroids of each cluster. (**B**) Mechanical pain differential (MPS-MPT) and NPS 3 (burning pain). (**C**) NPS 3 (burning pain) and H-reflex rate-dependent depression [HRDD (H2-5)] at 3 Hz. (**D**) Heat pain differential (HPT-WDT) and H-reflex rate-dependent depression [HRDD (H2-5)] at 3 Hz.

Amongst the pain group, MPS-MPT was also higher in patients with the most impaired HRDD compared to those with relatively unimpaired HRDD, although this was not significant (*P* = 0.0589) (Fig. 6B). Both within the pain cohort and across the whole patient group, increasing MPS-MPT values were associated with increasing impairment of HRDD [pain cohort: MPS-MPT and HRDD at 3 Hz (*r*_s_ = 0.414, *P* = 0.028) (Fig. 7A); all patients: 1 Hz (*r*_s_ = 0.232, *P* = 0.039) and 3 Hz (*r*_s_ = 0.231, *P* = 0.047) (Supplementary Table 1)]. Although not significant following Bonferroni correction, a greater impairment of HRDD is associated with higher ratings for pinprick-evoked pain relative to detection thresholds for pinprick pain.

### Cold pain differential = cold pain threshold − cold detection threshold

There was no significant difference in CPT-CDT between the pain and no pain groups. Amongst the pain group, there was no significant difference in CPT-CDT between patients with the most impaired HRDD compared to those with unimpaired HRDD (Fig. 6B). There was no correlation between CPT-CDT and HRDD at any frequency. However, within the pain group, a gain of function in CPT was associated with increasing impairment of HRDD: 1 Hz (*r*_s_ = 0.377, *P* = 0.048); 2 Hz (*r*_s_ = 0.423, *P* = 0.031); however, this was not significant following Bonferroni correction (Supplementary Table 2).

### Heat pain differential = heat pain threshold − warm detection threshold

There was no significant difference in HPT-WDT between the pain and no-pain groups. Amongst the pain group HPT-WDT was significantly higher in patients with the most impaired HRDD compared to those with relatively unimpaired HRDD (*P* = 0.0226) (Fig. 6B). Within the pain cohort, increasing HPT-WDT values were associated with increasing impairment of HRDD (HPT-WDT and HRDD at 2 Hz (*r*_s_ = 0.552, *P* = 0.003) and 3 Hz (*r*_s_ = 0.524, *P* = 0.007) (Fig. 7D), although not significant following Bonferroni correction. Therefore, amongst patients with painful DPN, a greater impairment of HRDD was associated with heat stimuli being felt as painful at lower temperatures relative to innocuous heat detection.

### Impairment of HRDD and accompanying relative mechanical hyperalgesia are associated with higher ratings of symptomatic burning pain

Within the cohort of patients with painful DPN, increasing impairment of HRDD and increasing values of MPS-MPT were associated with increasing reported scores for burning pain on the NPS questionnaire: HRDD at 3 Hz (*r*_s_ = 0.389, *P* = 0.037) (Fig. 7C); MPS-MPT (*r*_s_ = 0.484, *P* = 0.002) (Fig. 7B). Although not significant following Bonferroni correction, these associations, along with the earlier described associations between HRDD and MPS-MPT, lend support to the presence of a mechanistically relevant relationship between these parameters. Furthermore, MPS-MPT showed a significant correlation with ratings for intensity of pain on the NPS (*r*_s_ = 0.547, *P* < 0.001) (Supplementary Table 2).

### Cluster analysis

The large number of parameters in the correlation analysis (*n* = 38), resulted in a Bonferroni corrected significance value *P* < 0.001, to account for multiple comparisons. This highly conservative correction runs the risk of a type 2 error and overlooking potential associations.

To further explore the phenotypic manifestations of spinal disinhibition, we used a *k*-means cluster analysis technique that separates data sets for maximal similarity within clusters and dissimilarity between clusters, using 26 parameters (HRDD/QST/NPS pain descriptors) for segregation into two clusters. Five parameters significantly discriminated between the two clusters: HRDD at 1, 2 and 3 Hz, MPS-MPT and HPT-WDT. NPS3 (burning pain) was close to being significant (Supplementary Table 4A).

Cluster 1 (*n* = 11) exhibited impaired HRDD at all three frequencies, along with a relatively high value for both MPS-MPT (high MPS relative to their MPT) and HPT-WDT (heat hyperalgesia relative to innocuous heat detection) and higher reported scores for burning pain (NPS3). Cluster 2 (*n* = 21) exhibited intact HRDD at all three frequencies, along with MPS-MPT and HPT-WDT values close to 0, indicating a lack of relative mechanical and thermal hyperalgesia respectively (Fig. 7A). The mean values for each parameter within the two clusters can be seen in the final cluster centres table (Supplementary Table 4B).

## Discussion

A current major goal of research in clinical pain is to develop individualized treatment strategies based on presumptive or identifiable mechanisms of pain to increase efficacy and reduce side effects. We have previously shown that cohorts of patients with painful DPN have impaired HRDD, a biomarker of spinal disinhibition, when compared to patients with painless DPN and control subjects.^22-24^ However, individual patients with painful DPN exhibit HRDD values that vary markedly. A major objective of the current study was to explore whether impairment in HRDD was associated with a distinct pain phenotype. By deep phenotyping of patients using neuropathy symptom and pain questionnaires combined with QST, we demonstrated that patients with painful DPN exhibiting the most impaired HRDD (and hence greater spinal disinhibition) showed (i) greater MPS, especially when compared to mechanical pain detection, (ii) relative heat hyperalgesia when compared to innocuous warm detection, and (iii) higher ratings of spontaneous burning pain. These initial findings raise the intriguing possibilities that not only this is impaired HRDD in painful DPN associated with a distinct pain phenotype but also this phenotype is mechanistically appropriate for spinal disinhibition.

In line with previous studies,^4,13^ the dominant QST profile for our patients with DPN was that of loss of function. Whilst only a small minority of patients had thermal threshold parameters outside the normal range, approximately one in five demonstrated loss of function for tests of mechanical sensation. Nerve conduction parameters significantly correlated with the multiple of QST *z*-scores indicating a relationship between large fibre neuropathy and loss of function. In addition, greater corneal nerve fibre loss was associated with thermal hypoesthesia and mechanical hypoalgesia. We have shown a less marked loss of function than previously demonstrated in large multi-centre sensory phenotyping studies in DPN,^4,13^ which most likely reflects the less severe neuropathy in our cohort of patients. Indeed, ∼50% of patients in the Pain in Neuropathy Study (PiNS) had absent sural sensory nerve action potentials. As we were investigating the role of HRDD, our recruitment deliberately targeted patients most likely to have an adequate H-reflex and hence less severe neuropathy. However, of note, the severity of neuropathy was comparable between painful and painless cohorts, allowing valid phenotype comparison between the groups.

A predicted impact of spinal disinhibition (impaired HRDD) is that a given peripheral input to the dorsal horn of the spinal cord will be less suppressed than in the normal state or even facilitated.

Within the painful DPN cohort, we found positive correlations between HRDD, MPS and differential scores for both mechanical and heat thresholds. Whilst correlation coefficients were moderate, this exploratory analysis reveals a consistent relationship suggesting that spinal disinhibition may be associated with a combined sensory detection loss and hyperalgesia profile. In support of these findings, cluster analysis revealed that the presence of a spinal disinhibition sub-group in which impaired HRDD is clustered with mechanical and heat differentials and burning pain.

Patients with painful DPN required greater pinprick force to detect a stimulus as painful. Accordingly, in the absence of central facilitation/lack of suppression, one might expect lower pain scores during the stimulus response function. However, in patients with the most impaired HRDD, all of whom had painful DPN, mechanical pain differentials were higher than in patients with the most efficient HRDD. These findings indicate that when patients with impaired HRDD detect a painful punctate mechanical stimulus, they rate that stimulus as more painful than patients do with preserved/relatively preserved spinal inhibition. It is important to note that ratings of pain intensity relative to stimulus detection were only tested in the mechanical domain and the equivalent tests for cold and heat pain were not performed. In this respect, future hypothesis-driven studies that compare thermal pain threshold and pain ratings as well as the mechanical differential will be of interest. However, despite having equivalent impairment in detection thresholds for innocuous heat, patients with painful DPN who had greater impairment of HRDD showed a relative gain in function for heat pain detection (i.e. a relative heat hyperalgesia) compared to patients with painful DPN and efficient HRDD. These initial findings, which are arguably akin to hyperpathia, indicate that patients with spinal disinhibition may have a pain phenotype consistent with spinal amplification/reduced suppression. Furthermore, these exploratory findings provide a potential mechanism by which patients with DPN and an apparent deafferented phenotype develop neuropathic pain that can be detected psychophysically. Defined as ‘a painful syndrome characterized by an abnormally painful reaction to a stimulus, especially a repetitive stimulus, as well as an increased threshold’ (IASP), hyperpathia is not easily captured with QST and therefore may be underestimated.^33^ Classification of patients based on their sensory profile or phenotype has previously defined three clusters: those with sensory loss, thermal hyperalgesia and mechanical hyperalgesia.^5^ However, it is likely that these groups are not distinct, with additional nuanced phenotypes to be further defined.^5,34^ Indeed, our cluster analysis did not discriminate between the two clusters (with and without impaired HRDD) based on any individual QST parameters. However, the mechanical and heat differential scores were significant segregators. Therefore, the current findings do reveal a consistent theme linking spinal disinhibition to a combined sensory detection loss and hyperalgesia profile. However, future larger scale studies that enable hierarchical cluster analysis of QST sensory profiles will be needed to test this hypothesis.^35^

Neuropathic pain and particular phenotypic profiles are likely to relate to a complex interplay in the balance between peripheral inputs and central processing. Impairment of HRDD was not associated with a sensory profile suggestive of an irritable nociceptor phenotype. Both within the painful DPN group and across all patients with DPN, there was no significant relationship between HRDD and magnitude of wind-up or between HRDD and conditioned pain modulation. Whilst this suggests the mechanisms underlying spinal disinhibition are not directly related to other spinal processes that putatively result in central pain amplification (wind-up/temporal facilitation or dysfunctional descending pain modulation), it does not exclude an interaction or competition between these mechanisms. For example, an appropriate descending inhibitory control signal acting on a disinhibited spinal cord dorsal horn could be rendered ineffective or even facilitate ascending nociceptive drive. Interestingly, in the current study, the level of conditioned pain modulation measured with a pressure algometer did not differ significantly between patients with DPN with and without pain. This is consistent with recent findings obtained utilizing mechanical test stimuli.^19^ Indeed, the latter study also showed that conditioned pain modulation was unexpectedly more efficient in patients with painful DPN when noxious heat was used as a test stimulus.^19^ Further work incorporating different measures of the descending pain modulation system will be required to explore these potential interactions. The circuitry and pharmacology of HRDD exhibits considerable complexity and can be modified by a number of factors.^36^ It is also possible that other neurophysiological processes within the spinal cord might act concomitantly to modify spinal disinhibition, HRDD and nociceptive signalling in the dorsal horn of the spinal cord. However, there is currently no evidence that potential candidates, such as loss of segmental inhibition due to alterations of primary afferent depolarization, are implicated in animal models of diabetic neuropathy, and recent evidence suggests any effect of primary afferent depolarization on HRDD to be minimal.^37^

Limitations of this study include the collection of pain ratings by a one-time assessment rather than in a diary. Furthermore, patients continued to take their anti-neuropathic pain medication that would be expected to impact on the pain ratings. Treatments with particular anti-neuropathic pain drugs could also differentially alter HRDD that could increase variability or, by normalizing HRDD, have a tendency to underestimate the initial level of spinal disinhibition.^38^ Moreover, we enrolled patients with mild/moderate DPN as patients with severe polyneuropathy are likely to have an absent or inadequate H-reflex preventing an assessment of HRDD.^39,40^ Finally, the study is cross-sectional in nature. Longitudinal studies will be needed to assess the role of spinal disinhibition in evolving pain phenotypes or in the transition to chronic neuropathic pain as well as for the systematic evaluation of the effects of anti-neuropathic pain medications on HRDD, pain and sensory phenotype.

Both HRDD and QST are non-invasive and potentially widely applicable and broadly applicable in a clinical setting. HRDD and the distinct QST profile could be utilized to identify patients with painful DPN in whom disinhibition is a primary mechanism. This could direct mechanism-led therapeutics and drug discovery. For example, pharmacological intervention studies in diabetic rodents using duloxetine normalize HRDD and diminish behavioural indices of pain.^22,41^ Furthermore, an initial study indicates that the degree of normalization of HRDD predicts a therapeutic response to gabapentin in patients with painful DPN.^38^

## Conclusion

In conclusion, we have demonstrated that patients with painful DPN have impairment of HRDD and therefore evidence of spinal disinhibition. Furthermore, our initial findings using detailed pain profiling have revealed that greater impairment of HRDD is associated with higher patient ratings for burning pain and a ‘hyperpathia’ type profile, characterized by a loss of function in mechanical and thermal detection but with relatively high pain sensitivity. Further investigations to confirm and expand these intriguing findings are needed including an exploration of the therapeutic implications of identifying impaired HRDD and the interactions of spinal disinhibition with other peripheral and centrally mediated mechanisms of pain.

## Supplementary Material

fcad051_Supplementary_Data

## Data Availability

Data sets are available from the corresponding author upon request.

## Abbreviations

CCM =: corneal confocal microscopy
DNS =: Diabetic Neuropathy Symptom score
DPN =: diabetic peripheral neuropathy
HRDD =: Hoffmann reflex rate-dependent depression
NPS =: Neuropathic Pain Scale
NSP =: Neuropathy Symptom Profile
QST =: Quantitative Sensory Testing
SFN-SIQ =: Small Fibre Neuropathy and Symptom Inventory Questionnaire
VAS =: Visual Analogue Scale
