# Supplementary material for: Spinal disinhibition: evidence for a hyperpathia phenotype in painful diabetic neuropathy
Source: Brain Commun. 2023 Feb 28;5(2):fcad051. doi: 10.1093/braincomms/fcad051 (PMC10016414; doi:10.1093/braincomms/fcad051)
Supplement: fcad051_Supplementary_Data [file fcad051_supplementary_data.pdf]

Supplementary Material

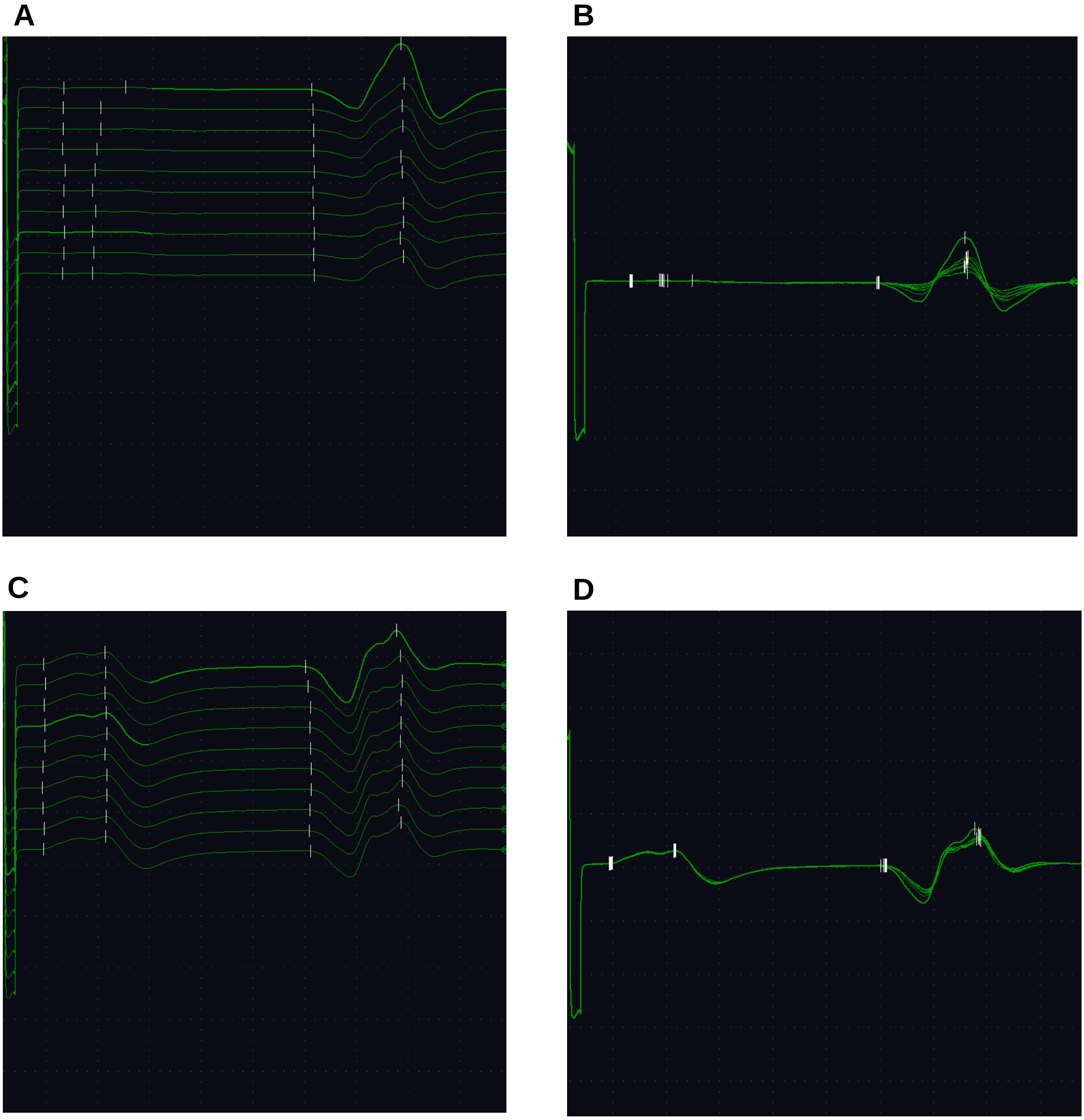

Supplementary Figure 1. Representative raw HRDD traces. (A) H-reflex trace at 1Hz in a patient with DPN without pain (B) H-reflex traces from (A) superimposed. (C) H-reflex trace at 1Hz in a patient with DPN with pain (D) H-reflex traces from (C) superimposed.

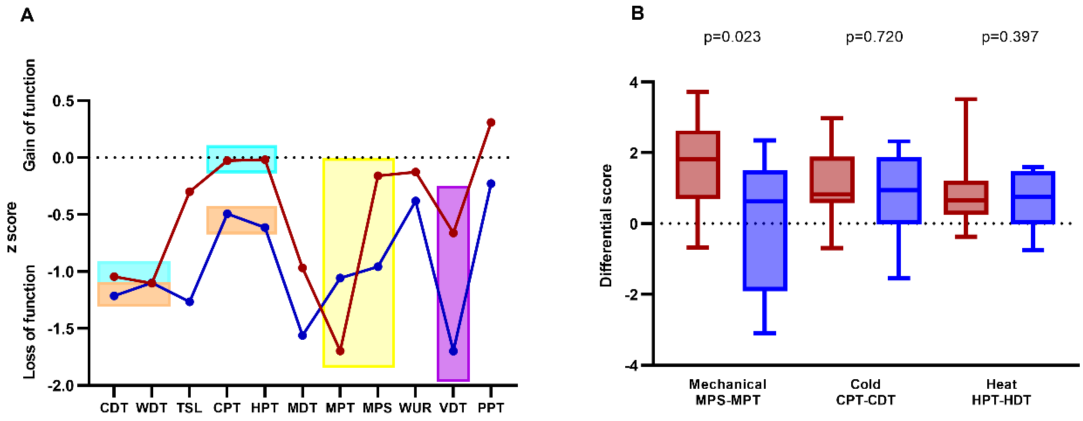

Supplementary Figure 2. Profile of patients with DPN with impaired and intact HRDD A. QST profile for patients with DPN with impaired (red circles/line) and intact (blue circles/line) HRDD. Orange box highlights z-scores for thermal detection and pain thresholds in patients with DPN and intact HRDD. Green box highlights z-scores for thermal detection and pain thresholds in patients with DPN and impaired HRDD. Yellow box highlights z-scores for mechanical pain threshold and sensitivity in patients with intact and impaired HRDD. Purple box highlights z-scores for vibration detection thresholds in patients with intact and impaired HRDD. **B.** Mechanical and thermal pain differentials in patients with impaired (red) and intact (blue) HRDD. Statistically significant p-values shown (unpaired t test). CDT, cold detection threshold; WDT, warm detection threshold; TSL, thermal sensory limen; CPT, cold pain threshold; HPT, heat pain threshold; MDT, mechanical detection threshold; MPT, mechanical pain threshold; MPS, mechanical pain sensitivity; WUR, wind-up ratio; VDT, vibration detection threshold; PPT, pressure pain threshold.

A	Cold Detection Threshold	Warm Detection Threshold	Thermal Sensory Limen	Cold Pain Threshold	Heat Pain Threshold	Pressure Pain Threshold	Mechanical Detection Threshold	Mechanical Pain Threshold	Mechanical Pain Sensitivity	Wind Up Ratio	Vibration Detection Threshold	MPS-MPT	CPT-CDT	HPT-WDT	Dynamic Mechanical Allodynia	Paradoxical Heat Sensations	Conditioned Pain Modulation
HRDD 1Hz	-0.016	-0.013	0.070	0.142	0.004	0.083	0.104	-0.204	0.005	0.113	0.130	0.232	0.090	0.131	0.086	-0.246	0.104
	0.892	0.911	0.557	0.221	0.973	0.473	0.361	0.069	0.963	0.320	0.253	0.039	0.442	0.259	0.443	0.028	0.448
HRDD 2Hz	-0.018	-0.017	0.050	0.104	-0.037	0.107	0.059	-0.092	0.059	-0.060	0.138	0.167	0.042	0.046	-0.097	-0.149	0.212
	0.879	0.888	0.687	0.389	0.761	0.374	0.619	0.437	0.620	0.613	0.246	0.155	0.727	0.704	0.406	0.206	0.125
HRDD 3Hz	-0.055	-0.071	-0.072	0.063	0.002	0.070	0.036	-0.162	0.074	0.149	0.074	0.231	0.083	0.185	0.011	-0.034	0.177
	0.649	0.557	0.568	0.605	0.987	0.563	0.759	0.168	0.533	0.209	0.533	0.047	0.497	0.124	0.927	0.776	0.210

B	HRDD 1Hz	HRDD 3Hz	Cold Detection Threshold	Warm Detection Thrshold	Thermal Sensory Limen	Cold Pain Threshold	Heat Pain Threshold	Presure Pain Threshold	Mechanical Detection Threshold	Mechanical Pain Threshold	Mechanical Pain Sensitivity	Wind Up Ratio	Vibration Detection Threshold	MPS-MPT	CPT-CDT	HPT-WDT	Dynamic Mechanical Allodynia	Paradoxical Heat Sensations	Conditioned Pain Modulation
Corneal Nerve Fibre Density	-0.082	-0.033	0.336	0.230	0.371	0.051	0.078	-0.044	0.108	0.274	0.112	-0.252	0.304	-0.116	-0.376	-0.203	-0.068	0.034	0.127
	0.494	0.791	0.002	0.038	0.001	0.647	0.484	0.696	0.335	0.013	0.324	0.024	0.006	0.301	0.000	0.067	0.544	0.761	0.335
Corneal Nerve Fibre Length	-0.017	-0.025	0.265	0.223	0.346	-0.049	0.077	-0.124	0.063	0.155	0.150	-0.271	0.128	0.001	-0.331	-0.208	-0.074	0.121	-0.084
	0.885	0.844	0.016	0.044	0.002	0.659	0.493	0.269	0.576	0.164	0.184	0.015	0.251	0.996	0.002	0.061	0.505	0.278	0.522
Corneal Nerve Branch Density	0.049	0.003	0.177	0.205	0.286	0.010	0.084	-0.091	0.137	0.126	0.321	-0.214	0.065	0.196	-0.232	-0.099	0.003	0.066	0.076
	0.696	0.981	0.128	0.078	0.016	0.933	0.476	0.442	0.242	0.282	0.005	0.069	0.582	0.091	0.045	0.398	0.978	0.574	0.587
Sural Nerve Amplitude	0.225	0.189	0.277	0.269	0.408	0.511	0.371	0.117	0.280	-0.015	0.302	-0.046	0.433	0.224	-0.034	0.275	-0.009	-0.253	0.072
	0.045	0.107	0.011	0.014	0.000	0.000	0.001	0.287	0.008	0.891	0.005	0.677	0.000	0.037	0.759	0.012	0.937	0.017	0.582
Sural Nerve Conduction Velocity	0.082	0.182	0.294	0.344	0.355	0.183	0.241	0.054	0.271	0.038	0.258	-0.118	0.289	0.135	-0.202	0.031	0.006	-0.118	0.046
	0.473	0.123	0.007	0.001	0.001	0.097	0.028	0.626	0.011	0.729	0.017	0.281	0.007	0.216	0.067	0.784	0.956	0.276	0.724
Peroneal Motor Amplitude	0.124	0.090	0.435	0.280	0.303	0.257	0.213	0.033	0.219	0.119	0.221	-0.180	0.428	0.065	-0.310	-0.016	-0.095	-0.069	0.131
	0.276	0.451	0.000	0.011	0.007	0.021	0.056	0.767	0.043	0.275	0.043	0.101	0.000	0.556	0.005	0.888	0.381	0.527	0.319
Peroneal Motor Conduction Velocity	0.137	0.031	0.430	0.387	0.515	0.322	0.211	-0.062	0.493	-0.014	0.281	-0.156	0.355	0.220	-0.242	-0.068	-0.154	-0.131	0.024
	0.232	0.794	0.000	0.000	0.000	0.004	0.060	0.584	0.000	0.901	0.010	0.159	0.001	0.045	0.030	0.547	0.157	0.232	0.855

C	HRDD 1Hz	HRDD 3Hz	Cold Detection Threshold	Warm Detection hresholdT	Thermal Sensory Limen	Cold Pain Threshold	Heat Pain Threshold	Presure Pain Threshold	Mechanical Detection Threshold	Mechanical Pain Threshold	Mechanical Pain Sensitivity	Wind Up Ratio	Vibration Detection Threshold	MPS-MPT	CPT-CDT	HPT-WDT	Dynamic Mechanical Allodynia	Paradoxical Heat Sensations	Conditioned Pain Modulation	Corneal Nerve Fibre Density	Corneal Nerve Fibre Length	Corneal Nerve Branch Density
Visual Analogue Score Current	0.552	0.403	-0.153	-0.081	-0.001	0.029	-0.044	0.188	-0.205	-0.248	-0.109	0.234	-0.172	0.243	0.150	0.058	0.115	-0.142	0.058	-0.033	0.017	0.123
	0.000	0.000	0.160	0.458	0.995	0.789	0.684	0.082	0.051	0.018	0.311	0.028	0.105	0.021	0.168	0.598	0.276	0.180	0.650	0.772	0.879	0.289
Visual Analogue Score Average	0.536	0.473	-0.205	-0.153	-0.083	-0.035	-0.088	0.145	-0.249	-0.268	-0.070	0.176	-0.170	0.263	0.174	0.087	0.067	-0.109	0.045	-0.104	-0.043	0.083
	0.000	0.000	0.058	0.161	0.458	0.749	0.421	0.179	0.017	0.010	0.513	0.098	0.110	0.012	0.109	0.427	0.527	0.304	0.725	0.353	0.703	0.478
Visual Analogue Score Maximum	0.542	0.475	-0.191	-0.142	-0.071	-0.028	-0.082	0.127	-0.236	-0.274	-0.078	0.197	-0.176	0.270	0.167	0.084	0.069	-0.106	0.037	-0.100	-0.039	0.084
	0.000	0.000	0.079	0.192	0.529	0.801	0.450	0.243	0.024	0.009	0.467	0.064	0.096	0.010	0.125	0.443	0.515	0.319	0.775	0.372	0.730	0.470
NPS 1 (Intensity)	0.500	0.475	-0.214	-0.178	-0.103	-0.009	-0.086	0.073	-0.294	-0.283	-0.077	0.171	-0.168	0.267	0.187	0.123	0.050	-0.097	0.029	-0.112	-0.079	0.004
	0.000	0.000	0.047	0.099	0.352	0.934	0.426	0.497	0.004	0.006	0.468	0.108	0.112	0.011	0.083	0.256	0.637	0.358	0.818	0.314	0.479	0.975
NPS 2 (Sharp)	0.359	0.305	-0.335	-0.196	-0.257	-0.207	-0.106	0.056	-0.319	-0.252	-0.068	0.105	-0.330	0.224	0.205	0.104	0.123	-0.067	0.008	-0.242	-0.154	0.013
	0.001	0.008	0.002	0.068	0.019	0.055	0.329	0.603	0.002	0.016	0.523	0.326	0.001	0.033	0.057	0.340	0.240	0.525	0.950	0.027	0.164	0.908
NPS (Burning)	0.502	0.557	-0.141	-0.144	-0.029	0.017	-0.036	0.055	-0.205	-0.207	0.028	0.176	-0.075	0.280	0.106	0.144	0.029	-0.093	0.027	-0.041	0.003	0.063
	0.000	0.000	0.192	0.182	0.791	0.877	0.743	0.612	0.050	0.048	0.794	0.097	0.478	0.007	0.327	0.183	0.781	0.379	0.830	0.715	0.976	0.588
NPS 4 (Dull)	0.391	0.414	-0.151	-0.050	-0.039	-0.025	-0.050	0.317	-0.020	-0.187	-0.210	0.126	0.021	0.074	0.178	0.054	0.111	-0.160	0.182	-0.016	-0.105	0.004
	0.000	0.000	0.164	0.642	0.723	0.819	0.648	0.003	0.850	0.074	0.047	0.238	0.847	0.485	0.099	0.619	0.288	0.127	0.149	0.887	0.343	0.975
NPS 5 (Cold)	0.169	0.258	-0.048	0.002	-0.030	0.052	0.124	0.275	-0.123	-0.061	0.160	0.068	-0.049	0.189	0.009	0.160	0.167	-0.056	-0.052	0.083	0.129	0.220
	0.131	0.025	0.660	0.983	0.785	0.635	0.251	0.009	0.243	0.565	0.131	0.525	0.647	0.073	0.934	0.139	0.109	0.598	0.685	0.456	0.246	0.056
NPS 6 (Sensitive)	0.286	0.224	0.009	0.045	0.067	0.189	0.163	0.271	-0.153	0.058	0.140	0.192	-0.191	0.087	0.052	0.198	0.113	-0.038	0.141	0.054	0.063	0.144
	0.010	0.054	0.934	0.678	0.547	0.079	0.132	0.011	0.146	0.583	0.190	0.070	0.070	0.410	0.633	0.066	0.280	0.720	0.267	0.627	0.570	0.215
NPS 7 (Itchy)	0.319	0.205	-0.187	-0.060	0.011	0.107	-0.010	0.216	-0.134	-0.133	-0.141	0.108	-0.229	0.050	0.197	0.039	0.159	-0.053	-0.094	0.073	0.146	0.129
	0.004	0.078	0.083	0.581	0.924	0.323	0.929	0.043	0.203	0.205	0.184	0.309	0.029	0.641	0.068	0.718	0.127	0.617	0.460	0.511	0.188	0.268
Diabetic Neuropathy Symptom Score	0.367	0.332	-0.289	-0.340	-0.239	-0.074	-0.258	0.064	-0.372	-0.269	-0.191	0.051	-0.299	0.152	0.257	0.059	0.142	-0.107	-0.038	-0.095	-0.036	-0.019
	0.001	0.006	0.010	0.002	0.039	0.516	0.022	0.573	0.000	0.013	0.086	0.651	0.006	0.169	0.022	0.603	0.194	0.333	0.777	0.414	0.760	0.875
Neuropathy Symptom Profile	0.146	0.266	-0.178	-0.310	-0.234	-0.073	-0.239	-0.025	-0.317	-0.205	-0.200	-0.038	-0.299	0.054	0.099	0.033	0.204	0.003	0.111	-0.074	-0.029	-0.045
	0.215	0.028	0.117	0.005	0.043	0.521	0.034	0.827	0.003	0.061	0.072	0.732	0.006	0.628	0.385	0.775	0.061	0.975	0.411	0.523	0.803	0.713

Supplementary Table 1. Correlations between **A:** HRDD, QST z-scores, QST differential scores and Conditioned Pain Modulation **B:** Large and small fibre parameters, HRDD, QST z-scores, QST differential scores and conditioned pain modulation **C:** Pain scores and descriptors, HRDD, QST z-scores and differential scores, conditioned pain modulation and corneal confocal parameters in patients with DPN. Data are Spearman correlations (rs) and significance (p). Significant correlations, $p<0.05$, are in boldface type.
HRDD, H-reflex rate dependent depression; NPS, neuropathy pain scale.

A	Visual Analogue Score Current	Visual Analogue Score Average	Visual Analogue Score Max	NPS 1 (Intensity)	NPS 2 (Sharp)	NPS 3 (Burning)	NPS 4 (Dull)	NPS 5 (Cold)	NPS 6 (Sensitive)	NPS 7 (Itchy)	Diabetic Neuropathy Symptom Score	Neuropathy Symptom Profile
HRDD 1Hz	-0.049	-0.146	-0.127	0.004	-0.326	0.129	-0.037	-0.201	-0.215	-0.045	0.042	-0.179
	0.79	0.426	0.488	0.984	0.069	0.483	0.841	0.27	0.236	0.807	0.612	0.864
HRDD 2Hz	-0.135	-0.073	-0.055	0.065	-0.317	0.289	-0.04	-0.133	-0.173	-0.087	-0.056	-0.158
	0.485	0.706	0.775	0.738	0.094	0.129	0.835	0.493	0.37	0.653	0.701	0.809
HRDD 3Hz	-0.197	0.032	0.044	0.175	-0.187	**0.389**	0.209	0.045	-0.114	-0.189	-0.06	-0.15
	0.307	0.87	0.819	0.364	0.331	**0.037**	0.276	0.816	0.555	0.325	0.818	0.499

B	HRDD 1Hz	HRDD 2Hz	HRDD 3Hz	Visual Analogue Score Current	Visual Analogue Score Average	Visual Analogue Score Max	NPS 1 (Intensity)	NPS 2 (Sharp)	NPS 3 (Burning)	NPS 4 (Dull)	NPS 5 (Cold)	NPS 6 (Sensitive)	NPS 7 (Itchy)	Diabetic Neuropathy Symptom Score	Neuropathy Symptom Profile
Cold Detection Threshold	0.028	0.113	0.068	0.095	-0.014	0.08	-0.271	**-0.527**	-0.07	-0.172	-0.028	0.199	-0.122	-0.273	-0.33
	0.886	0.582	0.748	0.613	0.939	0.667	0.134	**0.002**	0.703	0.346	0.877	0.276	0.506	0.138	0.069
Warm Detection Threshold	-0.207	-0.088	-0.061	0.192	-0.034	0.038	-0.285	-0.296	-0.099	-0.034	0.084	0.215	0.131	-0.33	-0.345
	0.291	0.67	0.771	0.3	0.855	0.84	0.115	0.1	0.591	0.853	0.649	0.238	0.475	0.07	0.057
Thermal Sensory Limen	0.017	0.063	0.018	0.258	0.037	0.118	-0.269	**-0.531**	-0.059	-0.063	-0.076	0.175	0.028	-0.212	**-0.443**
	0.935	0.768	0.937	0.185	0.853	0.55	0.158	**0.003**	0.763	0.746	0.695	0.365	0.885	0.279	**0.018**
Cold Pain Threshold	**0.377**	**0.423**	0.269	0.013	-0.151	-0.111	-0.151	**-0.607**	0.019	-0.051	0.165	0.217	0.244	-0.031	-0.23
	**0.048**	**0.031**	0.194	0.946	0.418	0.552	0.409	**0**	0.918	0.782	0.366	0.232	0.179	0.867	0.213
Heat Pain Threshold	-0.04	0.143	0.151	0.138	0.006	0.047	-0.245	-0.256	-0.003	-0.085	0.209	0.23	0.141	-0.294	**-0.409**
	0.84	0.484	0.471	0.46	0.973	0.803	0.177	0.157	0.988	0.642	0.25	0.205	0.442	0.109	**0.022**
Pressure Pain Threshold	-0.062	-0.047	0.112	0.251	0.157	0.056	-0.142	-0.144	-0.109	**0.36**	0.32	0.303	0.25	-0.011	-0.201
	0.749	0.82	0.587	0.165	0.389	0.763	0.43	0.425	0.547	**0.039**	0.07	0.086	0.161	0.952	0.269
Mechanical Dain Threshold	0.043	0.08	0.142	-0.193	-0.307	-0.232	**-0.356**	**-0.369**	-0.06	0.14	-0.112	-0.028	0.05	**-0.454**	**-0.474**
	0.819	0.687	0.47	0.266	0.073	0.18	**0.033**	**0.027**	0.726	0.416	0.514	0.87	0.77	**0.006**	**0.004**
Mechanical Pain Threshold	-0.194	0.008	-0.043	-0.153	-0.042	-0.091	**-0.364**	-0.226	-0.108	-0.071	0.061	0.296	0.056	-0.185	-0.195
	0.297	0.968	0.829	0.379	0.81	0.603	**0.029**	0.186	0.531	0.679	0.722	0.079	0.747	0.287	0.261
Mechanical Pain Sensitivity	0.169	0.342	**0.391**	-0.048	0.229	0.182	0.24	-0.007	**0.352**	-0.211	0.321	**0.366**	-0.009	-0.267	**-0.374**
	0.371	0.075	**0.04**	0.791	0.2	0.311	0.171	0.971	**0.041**	0.23	0.064	**0.033**	0.959	0.133	**0.032**
Wind Up Ratio	0.017	-0.092	-0.091	0.213	0.018	0.153	0.009	-0.051	0.158	-0.043	-0.023	0.168	0.091	0.078	-0.002
	0.929	0.642	0.644	0.227	0.919	0.388	0.959	0.771	0.366	0.805	0.896	0.336	0.602	0.663	0.99
Vibration Detection Threshold	0.18	0.208	0.363	-0.297	-0.178	-0.216	-0.308	**-0.543**	-0.115	0.184	-0.052	-0.25	**-0.378**	**-0.444**	**-0.367**
	0.342	0.298	0.063	0.088	0.315	0.221	0.072	**0.001**	0.511	0.29	0.766	0.147	**0.025**	**0.009**	**0.033**
MPS-MPT	0.227	0.276	**0.408**	0.202	0.252	0.277	**0.547**	0.23	**0.484**	-0.046	0.223	0.057	-0.021	-0.026	-0.088
	0.212	0.147	**0.028**	0.238	0.138	0.102	**0**	0.17	**0.002**	0.787	0.185	0.739	0.904	0.882	0.609
CPT-CDT	0.264	0.231	0.211	-0.116	-0.063	-0.123	0.199	0.209	-0.008	0.243	-0.012	-0.223	0.08	0.338	0.186
	0.175	0.257	0.312	0.534	0.738	0.51	0.275	0.252	0.965	0.181	0.95	0.221	0.332	0.063	0.317
HPT-WDT	0.328	**0.552**	**0.524**	-0.123	0.016	-0.001	0.072	0.008	0.188	-0.041	0.196	0.164	-0.182	-0.065	-0.112
	0.088	**0.003**	**0.007**	0.511	0.933	0.994	0.695	0.964	0.303	0.823	0.283	0.371	0.661	0.729	0.548
Dynamic Mechanical Allodynia	-0.072	-0.214	-0.294	-0.073	-0.095	0.093	-0.068	0.137	-0.295	0.006	0.075	0.157	0.296	-0.048	0.038
	0.696	0.266	0.121	0.669	0.583	0.588	0.687	0.418	0.076	0.974	0.659	0.355	0.076	0.78	0.825
Paradoxical Heat Sensations	-0.183	-0.064	-0.017	0.236	0.175	0.022	0.164	0.169	0.106	-0.109	0.053	0.017	-0.05	-0.082	0.092
	0.317	0.741	0.932	0.159	0.306	0.899	0.331	0.318	0.534	0.522	0.754	0.922	0.769	0.635	0.593

C	HRDD 1Hz	HRDD 2Hz	HRDD 3Hz	Visual Analogue Score Current	Visual Analogue Score Average	Visual Analogue Score Max	NPS 1 (Intensity)	NPS 2 (Sharp)	NPS 3 (Burning)	NPS 4 (Dull)	NPS 5 (Cold)	NPS 6 (Sensitive)	NPS 7 (Itchy)	DNS Total	NSP
Sural Nerve Amplitude	0.25	0.354	**0.426**	0.005	-0.054	-0.172	-0.164	**-0.395**	0.086	-0.042	0.192	0.035	-0.137	-0.066	-0.133
	0.167	0.059	**0.043**	0.981	0.781	0.373	0.331	**0.016**	0.611	0.806	0.255	0.836	0.418	0.702	0.438
Sural Nnerve Conduction Velocity	0.162	0.249	0.181	0.203	0.219	0.188	0.087	-0.081	0.01	0.128	-0.026	0.019	0.06	**-0.363**	-0.219
	0.384	0.192	0.357	0.301	0.264	0.337	0.613	0.639	0.952	0.456	0.88	0.911	0.728	**0.032**	0.207
Peroneal Motor Nerve Amplitude	0.12	0.311	0.27	-0.155	-0.113	-0.256	-0.276	**-0.399**	-0.067	0.202	0.12	-0.203	-0.193	-0.224	-0.042
	0.521	0.107	0.164	0.421	0.559	0.179	0.109	**0.018**	0.703	0.244	0.491	0.243	0.267	0.203	0.814
Peroneal Motor Nerve Conduction	0.162	0.313	0.19	0.068	0.032	0.067	-0.048	-0.319	0.098	0.193	-0.135	-0.051	-0.045	-0.156	-0.223
	0.392	0.112	0.343	0.731	0.871	0.733	0.788	0.066	0.58	0.274	0.446	0.776	0.8	0.378	0.206
Corneal Nerve Fibre Density	-0.141	0.058	0.027	-0.143	-0.162	0.008	-0.336	**-0.426**	-0.125	0.034	0.196	0.107	0.123	0.002	-0.09
	0.492	0.787	0.902	0.45	0.4	0.967	0.07	**0.019**	0.51	0.857	0.3	0.573	0.517	0.993	0.643
Corneal Nerve Fibre Length	-0.068	0.184	0.081	0.113	0.076	0.205	-0.024	-0.151	0.103	-0.205	0.24	0.15	0.331	0.161	0.119
	0.741	0.389	0.713	0.553	0.695	0.285	0.902	0.425	0.587	0.277	0.202	0.428	0.074	0.403	0.538
Corneal Nerve Branch Density	-0.002	0.223	0.041	0.153	0.17	0.236	0.118	0.05	0.174	-0.143	0.269	0.124	0.261	-0.181	0.012
	0.991	0.294	0.854	0.42	0.377	0.218	0.55	0.799	0.375	0.469	0.166	0.531	0.18	0.367	0.953

Supplementary Table 2. Correlations between **A:** HRDD, pain scores and pain descriptors **B:** QST z-scores, HRDD, pain scores and pain descriptors **C:** Large and small fibre neuropathy parameters, HRDD, pain scores and pain descriptors in the painful DPN cohort.
Data are Spearman correlations (rs) and significance (p). Significant correlations, $p<0.05$, are in boldface type. HRDD, H-reflex rate dependent depression; NPS, neuropathy pain scale

	Painful DPN with impaired HRDD (n=11) Group mean	Painful DPN with intact HRDD (n=11) Group mean	• Difference between means ± SEM<br>• 95% confidence interval<br>• R squared (eta squared)
**Cold Detection Threshold (z-score)**	-1.045	-1.213	-0.1682 ± 0.5828<br>-1.384 to 1.048<br>0.004147
**Warm Detection Threshold (z-score)**	-1.104	-1.098	0.005455 ± 0.4862<br>-1.009 to 1.020<br>6.294e-006
**Thermal Sensory Limen (z-score)**	-0.299	-1.265	-0.9659 ± 0.6568<br>-2.341 to 0.4087<br>0.1022
**Cold Pain Threshold (z-score)**	-0.082	-0.662	-0.5798 ± 0.3723<br>-1.365 to 0.2057<br>0.1249
**Heat Pain Threshold (z-score)**	-0.033	-0.745	-0.7117 ± 0.8442<br>-2.493 to 1.069<br>0.04012
**Mechanical Detection Threshold (z-score)**	-0.946	-1.439	-0.4925 ± 0.6947<br>-1.952 to 0.9671<br>0.02716
**Mechanical Pain Threshold (z-score)**	-1.846	-1.830	0.01636 ± 0.9021<br>-1.879 to 1.912<br>1.828e-005
**Mechanical Pain Sensitivity (z-score)**	-0.300	-1.791	-1.491 ± 0.9209<br>-3.434 to 0.4517<br>0.1336
**Wind Up Ratio (z-score)**	-0.129	-0.076	0.05334 ± 0.5389<br>-1.079 to 1.186<br>0.0005439
**Vibration Detection Threshold (z-score)**	-0.434	-1.898	-1.463 ± 1.086<br>-3.754 to 0.8274<br>0.09654
**Pressure Pain Threshold (z-score)**	0.285	0.468	0.1832 ± 0.4640<br>-0.7916 to 1.158<br>0.008589
**MPS-MPT**	1.611	0.099	-1.513 ± 0.6200<br>-2.798 to -0.2269<br>0.2130
**CPT-CDT**	1.123	0.386	-0.7369 ± 0.6513<br>-2.125 to 0.6512<br>0.07865
**HPT-WDT**	1.321	-0.022	-1.343 ± 0.5286<br>-2.470 to -0.2168<br>0.3010

Supplementary Table 3. Quantitative sensory testing in patients with painful DPN and impaired / intact HRDD. Unpaired t-test: 95% confidence interval and effect size are reported.

ANOVA

	Cluster		Error			
	Mean Square	df	Mean Square	df	F	Sig.
HRDD1	10081.505	1	191.747	30	52.577	<.001
HRDD2	15024.248	1	302.171	30	49.721	<.001
HRDD3	11471.802	1	316.580	30	36.237	<.001
MPSMPT	14.851	1	3.016	30	4.924	.034
CPTCDT	3.521	1	1.436	30	2.452	.128
HPTWDT	5.428	1	.925	30	5.866	.022
CDT	.524	1	1.688	30	.310	.582
WDT	1.242	1	1.208	30	1.028	.319
TSL	1.828	1	1.614	30	1.133	.296
CPT	1.328	1	.664	30	2.002	.167
HPT	1.477	1	2.631	30	.562	.459
PPT	.913	1	1.056	30	.865	.360
MDT	.053	1	1.884	30	.028	.867
MPT	5.918	1	2.960	30	1.999	.168
MPS	2.019	1	3.384	30	.597	.446
WUR	.048	1	1.235	30	.039	.845
VDT	.384	1	4.422	29	.087	.770
DMS	.902	1	.882	30	1.023	.320
PHS	.455	1	.447	30	1.017	.321
NPS1	7.535	1	7.866	30	.958	.336
NPS2	5.466	1	11.692	30	.467	.499
NPS3	40.035	1	10.399	30	3.850	.059
NPS4	1.195	1	7.389	30	.162	.690
NPS5	2.923	1	8.285	30	.353	.557
NPS6	1.637	1	8.508	30	.192	.664
NPS7	.977	1	10.600	30	.092	.763

Final Cluster Centers

	Cluster	
	1	2
HRDD1	89.35	51.98
HRDD2	84.33	38.70
HRDD3	81.14	41.28
MPSMPT	1.81	.38
CPTCDT	.87	.17
HPTWDT	1.12	.26
CDT	-.96	-.69
WDT	-1.13	-.71
TSL	-.28	-.78
CPT	-.09	-.52
HPT	.00	-.46
PPT	.43	.08
MDT	-1.07	-1.16
MPT	-2.09	-1.18
MPS	-.28	-.80
WUR	-.05	-.14
VDT	-.66	-.90
DMS	.07	.42
PHS	.27	.52
NPS1	5.55	4.52
NPS2	3.27	4.14
NPS3	6.55	4.19
NPS4	2.45	2.05
NPS5	1.64	1.00
NPS6	2.00	2.48
NPS7	3.27	2.90

Supplementary Table 4. K-means cluster analysis **A:** ANOVA table **B:** Final cluster centres.
